# Cross Talk between MarR-Like Transcription Factors Coordinates the Regulation of Motility in Uropathogenic Escherichia coli

**DOI:** 10.1128/IAI.00338-18

**Published:** 2018-11-20

**Authors:** Courtney L. Luterbach, Harry L. T. Mobley

**Affiliations:** a Department of Microbiology and Immunology, University of Michigan Medical School, Ann Arbor, Michigan, USA; The University of Texas at Austin

**Keywords:** UPEC, adherence, fimbriae, flagellar motility, gene regulation

## Abstract

The MarR-like protein PapX represses the transcription of the flagellar master regulator genes *flhDC* in uropathogenic Escherichia coli (UPEC), the primary cause of uncomplicated urinary tract infections (UTIs). PapX is encoded by the *pap* operon, which also encodes the adherence factors termed P fimbriae. Both adherence and motility are critical for productive colonization of the urinary tract. However, the mechanisms involved in coordinating the transition between adherence and motility are not well characterized. UPEC strain CFT073 carries both *papX* and a homolog, *focX*, located in the *foc* operon encoding F1C fimbriae. In this study, we characterized the dose effects of “X” genes on flagellar gene expression and cross talk between *focX* and *papX*. We found that both FocX and PapX repress *flhD* transcription. However, we determined that the Δ*papX* mutant was hypermotile, while the loss of *focX* did not affect motility. We further investigated this phenotype and found that FocX functions as a repressor of *papX*. Additionally, we identified a proximal independent promoter upstream of both *focX* and *papX* and assessed the expression of *focX* and *papX* during culture in human urine and on LB agar plates compared to LB medium. Finally, we characterized the contributions of PapX and FocX to fitness in the ascending murine model of UTI and observed a subtle, but not statistically significant, fitness defect in colonization of the kidneys. Altogether, these results expand our understanding of the impact of carrying multiple X genes on the coordinated regulation of motility and adherence in UPEC.

## Abstract (precis)

The MarR-like protein PapX represses the transcription of the flagellar master regulator genes *flhDC* in uropathogenic Escherichia coli (UPEC), the primary cause of uncomplicated urinary tract infections (UTIs). PapX is encoded by the *pap* operon, which also encodes the adherence factors termed P fimbriae.

## INTRODUCTION

Escherichia coli, a common, and typically commensal, bacterial species, regularly colonizes the human gastrointestinal tract (1, 2). Some strains of E. coli, broadly referred to as extraintestinal pathogenic E. coli (ExPEC), are equipped with virulence and accessory genes that are lacking from their commensal counterparts and promote infections outside the intestine, including the urinary tract (3). Uropathogenic E. coli (UPEC) strains are the primary cause of approximately 80% of all community-acquired urinary tract infections (UTIs), which are a pervasive and costly public health burden afflicting approximately one-half of all women and one-fifth of men at least once in their lifetime (4, 5). Most UTIs are established when bacteria contaminate the periurethral area and migrate to the bladder via the urethra. While many UTIs are self-limiting and resolve within a few days, in some cases, UPEC may further ascend via the ureters and cause a more severe secondary infection in the kidneys, called pyelonephritis, which increases the risk of renal scarring, sepsis, and death (6).

Extracellular polymeric filaments called flagella promote swimming motility in bacteria and facilitate UPEC ascension of the urethra and ureters during UTIs (7, 8). While the ability to produce flagella is not required for colonization of the urinary tract, strains capable of flagellum-mediated motility persist longer within the bladder and kidneys and colonize to higher levels (9, 10). Moreover, expression of *fliC*, the subunit that polymerizes to form the flagellum, coincides with ascension of the urinary tract and colonization of the kidneys in the murine model of UTI (11). However, *fliC* is poorly expressed in UPEC strains isolated from the urine of women experiencing acute cystitis as well as during *in vitro* culture in human urine (12, 13). Additionally, flagella are energetically costly to produce, and monomeric FliC can be detected by Toll-like receptor 5 (TLR5) found on the uroepithelium (14, 15). Thus, expression of flagella during UTI may be detrimental for UPEC survival and contribute to the transient expression of flagellar genes observed during infection (11, 16).

During a UTI, bacteria transition between motile and adherent states through coordinated cross talk between genes encoding flagella and adherence factors, termed fimbriae (11, 17, 18). Adherence of bacteria to host cells is critical for colonization of the urinary tract, and UPEC isolates are more likely to encode fimbriae that bind to cells found within the urinary tract (19–21). UPEC strain CFT073 encodes 12 fimbriae, including F1C and two separate P fimbriae (22). F1C fimbriae, encoded by the *foc* operon, bind glycolipids found on the kidney epithelium and endothelium, and cystitis UPEC isolates were more likely than fecal E. coli isolates to encode F1C fimbriae (23–25). Similarly, pyelonephritis-associated pili (Pap), or P fimbriae, bind the P blood group antigen enriched on human kidney epithelial cells and erythrocytes, and UPEC strains harboring the *pap* operon are more likely to cause pyelonephritis (26, 27).

Differing from most fimbrial operons, only one of the two *pap* operons and the single *foc* operon carry a 3′-terminal gene encoding a MarR-like transcription factor, PapX or FocX, respectively. MarR-like proteins share a winged helix-turn-helix structure (wHTH), bind as dimers to palindromic DNA sequences, and have been shown to mediate the regulation of numerous genes, including those involved in resistance to antibiotics, oxidative stress, and low pH as well as motility (28–31). We have previously shown that PapX binds to a 29-bp palindromic DNA sequence centered 410 bp upstream of the *flhDC* translational start site, encoding the master transcriptional regulator FlhD_4_C_2_ of flagellar gene expression. Overproduction of PapX represses the transcription of *flhD* and subsequently reduces swimming motility (17, 32, 33). Consistent with our findings in CFT073, *papX* was also identified in a transposon directed insertion sequencing (TraDIS) screen for genes affecting motility in E. coli strain EC958 (34). Thus, PapX is directly involved in regulatory cross talk between genes associated with adherence and motility.

FocX shares 96.7% amino acid sequence identity with PapX and therefore is predicted to also function as a repressor of motility. However, the function of FocX in UPEC has not been well characterized, and the impact of encoding multiple homologous “X” proteins on motility in UPEC is poorly understood. In this study, we found that both PapX and FocX repress *flhD* expression as well as swimming motility when overproduced. However, we have discovered that FocX can also repress the expression of *papX* and that cross talk between “X” genes affects motility. Additionally, we characterized a proximal promoter for *focX* and *papX*, suggesting that *focX* and *papX* can be expressed independently from their respective fimbrial operons. However, we found that the expression of *papX* positively correlated with *pap* expression during *in vitro* culture on LB agar plates. Furthermore, we assessed the relative fitness of either the single Δ*papX* or the double Δ*focX* Δ*papX* mutant compared to the wild type by *in vivo* competitive cochallenge in CBA/J mice and observed a slight, but not statistically significant, decrease in kidney colonization. Since UPEC isolates are more likely than commensal strains to carry at least two X genes, investigating the interactions between PapX and FocX is imperative to understand how UPEC regulates motility and responds to environmental signals within the urinary tract.

## RESULTS

### PapX and FocX share high sequence and structural similarities.

**FIG 1:**
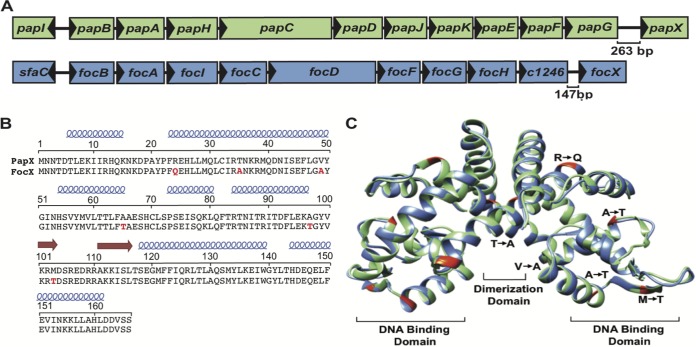
PapX and FocX share high sequence and structural homology. (A) Schematic of the *pap1* and *foc* operons in CFT073. (B) The amino acid sequences of PapX and FocX were manually aligned, and residues in red signify differences in FocX compared to PapX. The predicted location of α-helices (blue) and β-sheets (red arrows) are shown above the amino acid sequence. (C) The predicted dimer structures of PapX (green) and FocX (blue) were created by using I-TASSER and aligned to the known structure of the dimer HucR by using Chimera (not shown). Amino acid differences between FocX and PapX are highlighted in red and labeled on one monomer.

The E. coli CFT073 genome harbors both the *pap* and *foc* operons, which encode the homologous MarR-like proteins PapX and FocX, respectively (Fig. 1A) (22). The predicted structures of PapX and FocX were created by using I-TASSER and were modeled as dimers based on the solved dimer structure of the MarR-like protein HucR (35). While MarR-like proteins contain a conserved wHTH DNA binding motif, the majority of these proteins share limited amino acid sequence identity (∼25 to 35%) (36). Despite being encoded by different fimbrial operons, PapX and FocX share high amino acid sequence identity (96.7%) (Fig. 1B) and predicted structural homology (Fig. 1C) (32). In MarR-like proteins, key structural motifs include the dimerization domain between subunits, the DNA recognition helices that bind palindromic DNA sequences within the major groove, and the wing domain that interacts with residues within the DNA minor groove (37, 38). Therefore, amino acid changes within these structural domains are more likely to alter DNA binding-site recognition and protein function. There are three amino acid differences within key structural areas between PapX and FocX: T35A within the dimerization domain, A97T within the DNA binding helix, and M103T within the wing domain. Therefore, while we predict that PapX and FocX share the same function based on their high structural and amino acid sequence similarities, it is not known if these substitutions affect protein function.

### Loss of *papX*, but not *focX*, increases swimming motility.

**FIG 2:**
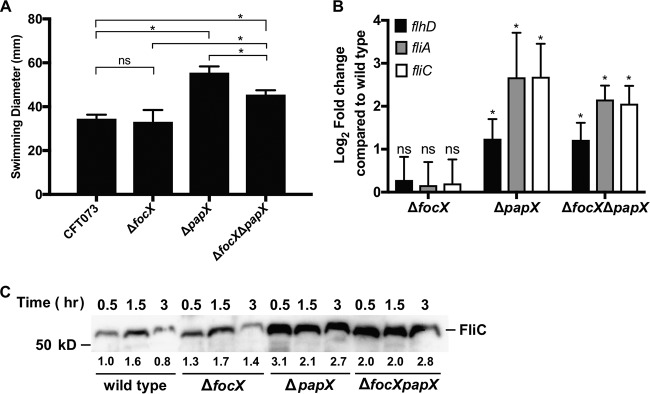
Effects of *focX* and *papX* expression on swimming motility. (A) Swimming diameter (millimeters) was measured for CFT073 and isogenic mutants after 16 to 18 h of incubation at 30°C. Data represent results for five biological replicates, with the error bars showing the standard deviations. Tukey’s multiple-comparison test following ANOVA was used for statistical analysis. *, *P < *0.05; ns, not significant. (B) qPCR of flagellar genes *flhD*, *fliA*, and *fliC* using cDNA collected from wild-type E. coli CFT073 and Δ*focX*, Δ*papX*, and Δ*focX* Δ*papX* constructs cultured in tryptone medium until the OD_600_ reached 0.3. Data represent the averages of results from three experiments. Standard deviations are shown, and Student’s *t* test was used for statistical analysis. *, *P < *0.05. No statistical difference was found between the Δ*papX* and Δ*papX* Δ*focX* strains using a Mann-Whitney test. (C) Immunoblotting to detect FliC levels from whole-cell lysates of wild-type CFT073 and the Δ*focX*, Δ*papX*, and Δ*papX* Δ*focX* constructs cultured in tryptone broth. Relative quantification of FliC (shown below the protein band) was obtained by using Image Lab 5.2.1 and represents the average fold change from two independent experiments compared to FliC levels of the wild type at 0.5 h and normalized to a conserved nonspecific band.

To determine the effects of PapX and FocX on motility, we measured the motility of the CFT073 wild-type, Δ*focX* mutant, Δ*papX* mutant, and Δ*focX* Δ*papX* double mutant strains using a swimming motility assay. We observed that the loss of *focX* did not affect swimming motility (Fig. 2A). In contrast, the loss of *papX* resulted in a hypermotile phenotype, and this result was consistent with previous studies showing that PapX represses motility (17, 32, 33, 39). Interestingly, the loss of both *focX* and *papX* resulted in an intermediate level of swimming that was significantly greater than that of the wild type but not as robust as that of the Δ*papX* mutant, suggesting that there may be cross talk between *focX* and *papX*.

E. coli strains differ in their capacities for motility due in part to heterogeneity in the presence of insertion sequence (IS) elements upstream of *flhDC* as well as variability in the encoded transcriptional regulators of flagellar genes (40–42). Therefore, to compare the functions of PapX in additional UPEC strains, we compared motility between the wild type and a Δ*papX* mutant in UPEC cystitis isolates F11 and HM69 (see Fig. S1 in the supplemental material). Both strains F11 and HM69 carry the *pap* operon, including *papX*, but not the *foc* operon. We observed that the deletion of *papX* resulted in a significant increase in motility compared to the wild type in F11 (121%) and HM69 (117%); however, the hypermotile phenotypes were not as robust as what was observed in CFT073 (161%) (Fig. 2A). Therefore, these data support that PapX functions through a conserved mechanism in UPEC but that the impact of PapX on motility is dependent on the strain background.

We have previously shown that PapX inhibits swimming motility by repressing the expression of *flhDC*, resulting in the downregulation of additional flagellar genes (32, 33, 39). To verify that the observed motility phenotypes correspond to changes in the expression of flagellar genes, we performed quantitative PCR (qPCR) to quantify the changes in the mRNA abundances of *flhD*, *fliA*, and *fliC* between CFT073 and the Δ*focX*, Δ*papX*, and Δ*focX* Δ*papX* constructs. RNA was collected from bacteria cultured in tryptone medium to an optical density at 600 nm (OD_600_) of 0.3. Growth conditions were chosen based on previous work demonstrating that the production of flagella in CFT073 is elevated during early-logarithmic-phase growth in tryptone medium (18). Consistent with our swimming results, the loss of *focX* did not alter the expression of *flhD*, *fliA*, or *fliC* (Fig. 2B). However, we observed a significant increase in the expression of *flhD*, *fliA*, and *fliC* in both the Δ*papX* (log_2_ fold change [FC], 1.2 to 2.7) and the double Δ*focX* Δ*papX* (log_2_ FC, 1.2 to 2.1) mutants compared to the wild type.

To determine if the qPCR results correlated with an increase in flagellum production, we performed immunoblotting to compare FliC (flagellin) levels between wild-type CFT073 and the Δ*focX*, Δ*papX*, and Δ*focX* Δ*papX* constructs. Bacteria were cultured in tryptone medium, and the levels of FliC were assessed from normalized whole-cell lysates collected at 0.5, 1.5, and 3 h, representing early-, mid-, and late-logarithmic-phase growth (Fig. S2). In wild-type CFT073, we observed a peak in FliC production at 1.5 h, and this result was consistent with data from previous studies characterizing the temporal production of FliC (18) (Fig. 2C). Additionally, we observed increases in FliC production in the Δ*papX* and Δ*focX* Δ*papX* constructs at all observed time points, which were quantified by densitometry based on the averages of data from two replicates. We did not detect an increase in FliC production in the Δ*focX* construct compared to the wild type. Therefore, these results show that the deletion of *papX*, compared to *focX*, has a great impact on swimming motility, flagellar gene expression, and FliC production in CFT073.

### Both PapX and FocX function as repressors of motility.

**FIG 3:**
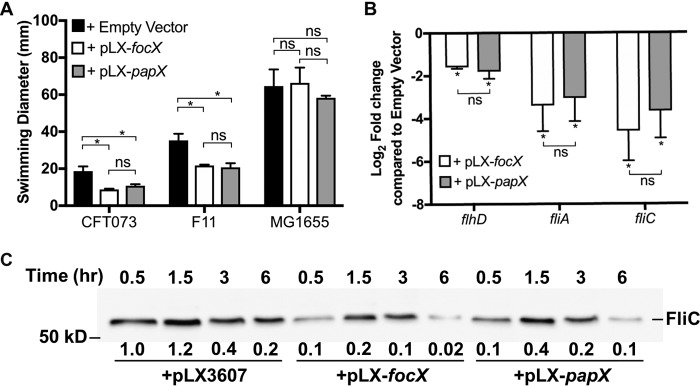
Expression of either *focX* or *papX* represses flagellar gene expression and motility. (A) Bars representing the average diameters (millimeters) of swimming motility of bacteria following 16 to 18 h of incubation at 30°C. Data represent results of five biological replicates, with the error bars showing the standard deviations. Tukey’s multiple-comparison test following ANOVA was used for statistical analysis. *, *P < *0.05; ns, not significant. (B) qPCR of the flagellar genes *flhD*, *fliA*, and *fliC* using cDNA synthesized from mRNA collected from E. coli CFT073 Δ*papX* Δ*focX* carrying pLX3607, pLX-*focX*, or pLX-*papX* cultured in tryptone medium until the OD_600_ reached 0.3. Data represent the averages of results from three experiments with standard deviations. Student’s *t* test was used for statistical analysis. (C) Immunoblotting to detect FliC levels from whole-cell lysates of wild-type CFT073 carrying pLX3607, pLX-*focX*, or pLX-*papX*. Relative quantification of FliC was obtained by using Image Lab 5.2.1 and represents the average fold change from two independent experiments compared to FliC levels of the Δ*papX* Δ*focX*/pLX3607 strain at 0.5 h and normalized to a conserved nonspecific band.

Based on the amino acid sequence and structural similarities between PapX and FocX, we predicted them to share the same function as transcriptional repressors of motility. However, compared to the wild type, the deletion of *focX* did not significantly increase swimming motility. Therefore, to assess PapX and FocX function independent of their native expression, we overexpressed *papX* and *focX* in CFT073 and performed a swimming motility assay (Fig. 3A). Compared to CFT073 carrying the empty vector pLX3607, expression of either *papX* (pLX-*papX*) or *focX* (pLX-*focX*) resulted in approximately a 50% reduction in swimming motility. In addition, complementation of *focX* and *papX* in the Δ*focX*, Δ*papX*, and Δ*focX* Δ*papX* mutants resulted in comparable levels of inhibition of motility (see Fig. S3 in the supplemental material). To assess whether this phenotype was specific to CFT073, we repeated this assay in the cystitis isolate F11 and observed a similar decrease in motility following the overproduction of either FocX or PapX. We have previously shown that the commensal E. coli K-12 MG1655 strain lacks the PapX binding site upstream of *flhDC*, and therefore, the overproduction of PapX does not reduce motility (32). To determine if the function of FocX also depends on the presence of the PapX binding site upstream of *flhDC*, we assessed the swimming motility of K-12 MG1655 carrying pLX3607, pLX-*papX*, or pLX-*focX*. We observed that the expression of either *papX* or *focX* did not decrease motility compared to the empty vector. Therefore, these data support that PapX and FocX share the same function as repressors of motility and that this mechanism is dependent on the presence of the PapX binding site upstream of *flhDC*.

To determine if the reduction in motility following the overexpression of *focX* or *papX* was a result of decreased expression of flagellar genes, we used qPCR to assess the gene expression levels of *flhD*, *fliA*, and *fliC* (class I, class II, and class III, respectively) in the Δ*focX* Δ*papX* double mutant carrying pLX3607, pLX-*papX*, or pLX-*focX*. We assessed flagellar gene expression in the double mutant background, versus the wild type, to eliminate any interference due to native levels of PapX and FocX. RNA was collected from bacteria cultured to an OD_600_ of 0.3 in tryptone medium. We found that the expression of either *papX* or *focX*, compared to the empty vector, resulted in a comparable decrease in the transcription of *flhD*, *fliA*, and *fliC* (Fig. 3B). To verify that our qPCR data correlated with a decrease in flagellin production, we performed immunoblotting for FliC using the Δ*focX* Δ*papX* mutant carrying pLX3607, pLX-*focX*, or pLX-*papX*. Bacteria were cultured in tryptone medium, and whole-cell lysates were collected at 0.5, 1.5, 3, and 6 h and normalized by the OD_600_. We observed that the overexpression of either *focX* or *papX* resulted in a decrease in FliC production at all observed time points compared to the empty vector (Fig. 3C). These data support that both FocX and PapX, when ectopically expressed, can repress *flhD*, resulting in decreased flagellar production and motility.

### *focX* and *papX* are transcribed from a proximal promoter.

**FIG 4:**
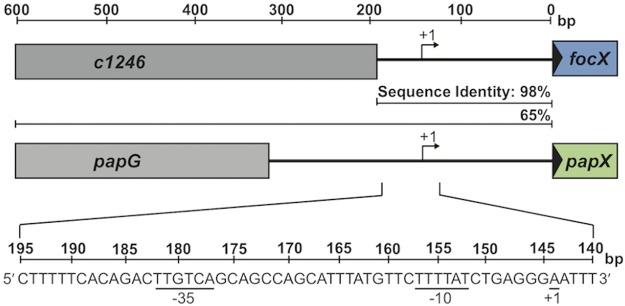
*focX* and *papX* are transcribed from a proximal promoter. Shown is a schematic of the transcriptional start sites of *focX* and *papX* identified by using 5′ RACE, followed by DNA sequencing. cDNA was synthesized from mRNA collected from either the Δ*papX* or Δ*focX* strain. A reference horizontal line measuring the DNA sequence length (base pairs) is included at the top. The transcriptional start site (+1) of either *focX* or *papX* is located 144 bp upstream of the initial ATG site of each gene. Putative −10 and −35 sites with similarity to the bacterial σ^70^ promoter are underlined. The DNA sequence identities (percent) of the upstream DNA sequences between *papX* and *focX* in CFT073 are shown for two DNA regions (200 bp and 600 bp) upstream of the ATG start site of *focX* or *papX*.

We have previously shown that *papX* is transcribed as part of the *pap* operon and have confirmed that *focX* is also transcribed as part of the *foc* operon (see Fig. S4 in the supplemental material) (33). However, the *papX* homolog *sfaX* is transcribed as part of the *sfa* operon as well as from an independent proximal promoter (43). Since the upstream DNA sequences of *papX*, *focX*, and *sfaX* share high sequence identity, *papX* and *focX* likely share a similar proximal promoter. We used 5′ rapid amplification of cDNA ends (RACE) to map the transcriptional start sites of both *papX* and *focX* to an adenosine residue located 144 bp upstream of their predicted ATG start codon (Fig. 4). We manually identified putative −10 and −35 regions separated by a 19-bp spacer that resemble the consensus sequence of a bacterial σ^70^ promoter. This site was shared between *papX* and *focX* but was located 44 bp upstream of the transcriptional start site identified for *sfaX* (43). The presence of an additional proximal promoter upstream of *focX* and *papX* suggests that both these genes can be transcribed independently and from their associated fimbrial operons.

### Expression of *papX* or *focX* trends with the expression of the preceding fimbrial operon.

**FIG 5:**
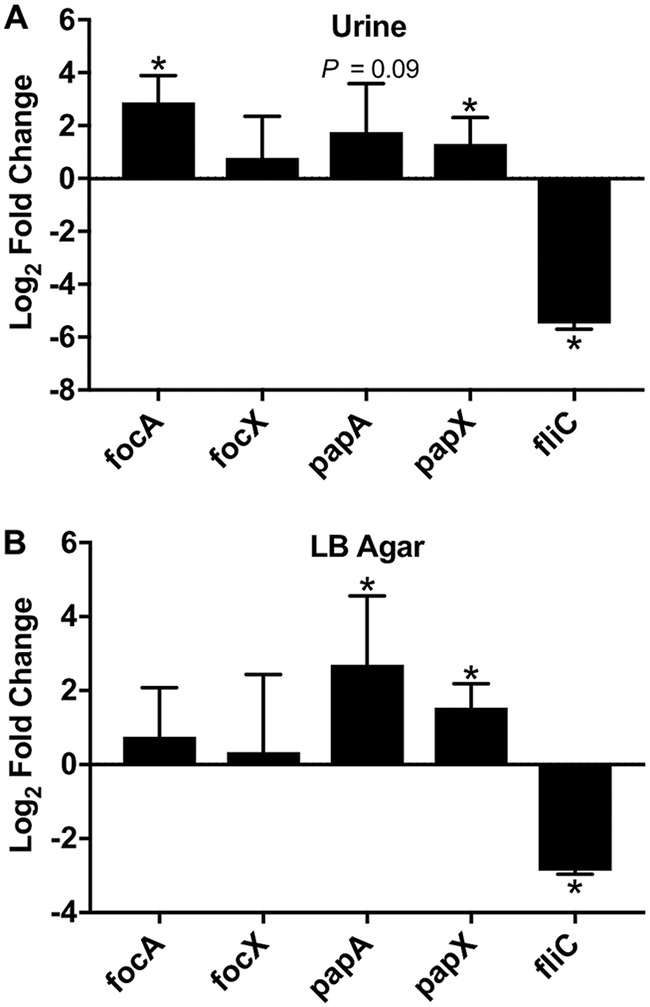
Expression of *foc*, *pap*, and *fliC* in strains grown in human urine or on LB agar. Shown are data from qPCR of *focA*, *focX*, *papA*, *papX*, and *fliC* using cDNA collected from wild-type E. coli CFT073 cultured in human urine until an OD_600_ of 0.3 was reached (A) or on LB agar for 24 h compared to cDNA collected from bacteria cultured to the mid-logarithmic growth phase in LB medium (B). Data represent the averages of results from three experiments, with error bars showing standard deviations. Student’s *t* test was used to calculate statistical significance. *, *P < *0.05.

Since *focX* and *papX* are also transcribed from an independent proximal promoter, we investigated the expression of *focX* and *papX* compared to the *foc* and *pap* operons, respectively, under different environmental conditions. Previous work by Hancock et al. determined that the *pap* and *foc* genes are upregulated in CFT073 during planktonic growth in human urine compared to morpholinepropanesulfonic acid (MOPS), but the expression of *focX* or *papX* was not investigated (44). Therefore, we used qPCR to quantify the expression of *papA*, *papX*, *focA*, *focX*, and *fliC* in wild-type CFT073 during culture to the mid-logarithmic growth phase in pooled human urine compared to LB medium. We included *fliC* to assess correlations of *focX* and *papX* with motility under these growth conditions. We found that culture in human urine compared to LB medium led to significant increases in the transcription of *focA* (log_2_ FC, 2.8) and *papX* (log_2_ FC, 1.3), but we did not detect a statistically significant change in *focX* or *papA* (Fig. 5A). Additionally, we observed a decrease in *fliC* expression (log_2_ FC, −5.48), and this finding was consistent with previous work showing decreased motility in UPEC strains cultured in human urine (13).

Previous studies have shown that the production of P fimbriae is elevated in the E. coli CFT073 strain cultured on LB agar compared to planktonic growth (45). Therefore, we assessed the expression of *papA*, *papX*, *focA*, *focX*, and *fliC* by qPCR following a 24-h incubation on LB agar plates at 37°C compared to culture in LB medium to the mid-logarithmic growth phase. Consistent with previous findings, we observed that the expression levels of *papA* (log_2_ FC, 2.7) and *papX* (log_2_ FC, 1.5) were significantly increased, but we did not observe a change in the expression of *focA* and *focX* (Fig. 5B). Additionally, *fliC* expression was decreased (log_2_ FC, −2.8) following incubation on LB agar plates. Overall, the relative expression levels of *focX* and *papX* were lower than that of the reference gene *gapA* under all three tested growth conditions (see Fig. S5 in the supplemental material).

### FocX functions as a repressor of *papX*.

**FIG 6:**
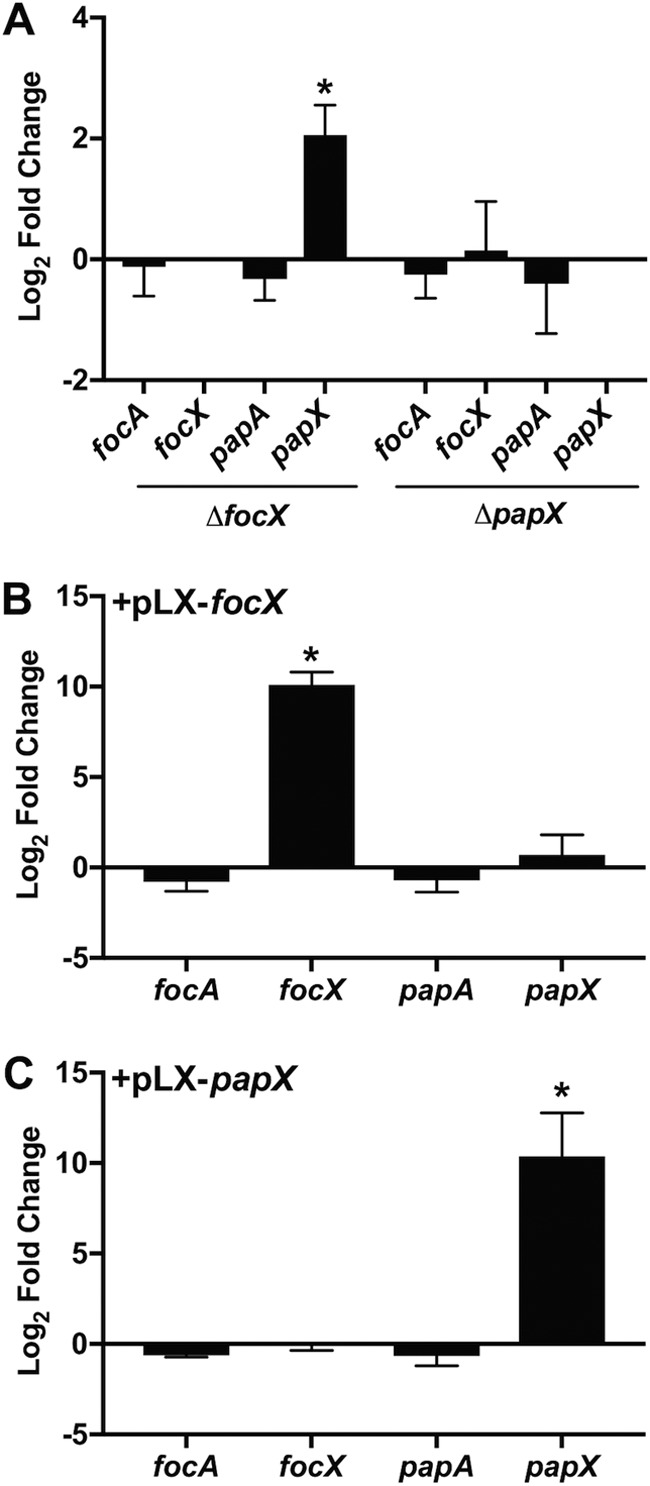
Loss of *focX* results in elevated *papX* expression. qPCR was performed to calculate the average (*n* = 3) log_2_ fold changes of mRNA abundances of *focA*, *focX*, *papA*, and *papX* using cDNA collected from the E. coli CFT073 wild-type, Δ*focX*, or Δ*papX* strain (A) or the wild type carrying pLX3607, pLX-*focX* (B), or pLX-*papX* (C) cultured in tryptone medium until an OD_600_ of 0.3 was reached. Errors are shown as standard deviations, and statistical significance was calculated using Student’s *t* test. *, *P < *0.05.

We have previously shown that the deletion of *papX* in CFT073 does not affect the production of P fimbriae; however, the effect of FocX on the expression of genes within the *foc* operon has not been characterized (33). Therefore, we performed qPCR to assess the changes in the gene expression levels of *focA*, *focX*, *papA*, and *papX* following the deletion of either *focX* or *papX*. cDNA was synthesized from mRNA collected from wild-type CFT073 and the Δ*focX* and Δ*papX* constructs cultured to the mid-logarithmic growth phase in tryptone medium. We found that the deletion of *focX* did not affect *focA* or *papA* expression but resulted in higher expression levels of *papX* (log_2_ FC, 2.06) (Fig. 6A). In contrast, the deletion of *papX* did not result in any significant changes in the gene expression of *focA*, *focX*, or *papA*. These data suggest that FocX functions as a repressor of *papX* and that the regulatory mechanism is not a result of decreased expression of the *pap* operon. Therefore, the regulation of *papX* may be occurring at the proximal *papX* promoter. However, we did not identify a binding site upstream of either *focX* or *papX* that matched the characterized PapX binding site upstream of *flhDC*, suggesting that regulation of *papX* may be indirect or occurs through a degenerate DNA binding site (32).

We also assessed the changes in the gene expression levels of *focA*, *focX*, *papA*, and *papX* in CFT073 when either *focX* or *papX* was ectopically expressed. cDNA was synthesized from mRNA collected from CFT073 harboring pLX3607, pLX-*focX*, or pLX-*papX* cultured to the mid-logarithmic growth phase in tryptone medium. We found that the production of FocX did not affect the expression of *focA*, *papA*, or *papX* (Fig. 6B). Similarly, the production of PapX did not impact the expression of *focA*, *focX*, or *papA*. Therefore, the function of FocX as a repressor of *papX* may be dependent on the protein concentration, as we did not observe a decrease in *papX* expression in response to high levels of FocX.

### *papX* provides a subtle fitness advantage in colonization of the kidneys during murine UTI.

**FIG 7:**
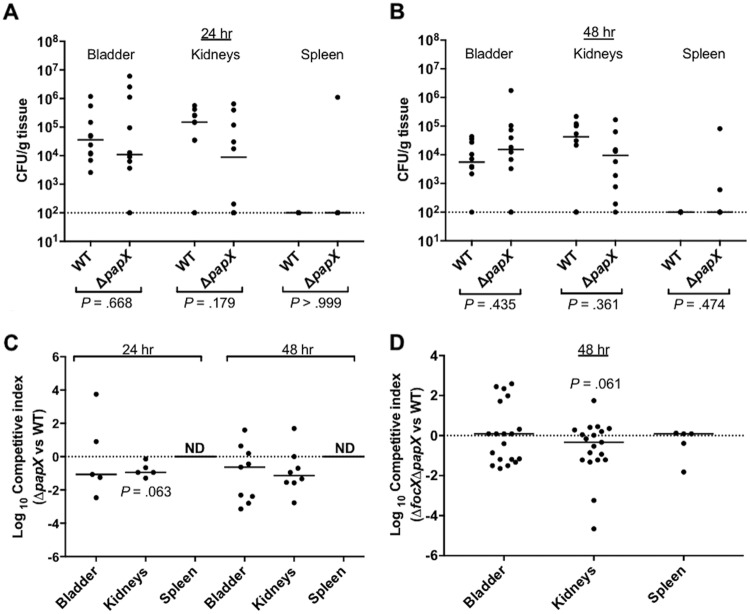
The contribution of PapX to *in vivo* colonization is subtle and observable only following intraurethral inoculation. (A and B) Independent challenges of CBA/J mice (*n* = 10) inoculated with a 10-μl suspension of 10^7^ CFU/ml of either wild-type (WT) CFT073 or the Δ*papX* mutant directly into the urethra. Mice were sacrificed at either 24 h (A) or 48 h (B) postinoculation, and the bladders, kidneys, and spleens were homogenized and plated to enumerate the bacterial load and are presented as CFU per gram of tissue. Statistical differences in colonization were determined by using a Mann-Whitney test. (C) Cochallenge infection of mice (*n* = 10) using a 1:1 mixture of the wild type and the Δ*papX* mutant. CBA/J mice were intraurethrally inoculated with a 10-μl suspension of a total of 10^7^ CFU/ml. Mice were sacrificed at either 24 h (*n* = 5) or 48 h (*n* = 10) postinoculation, and the bladders, kidneys, and spleens were homogenized and plated to enumerate the bacterial load. Relative fitness is presented as a competitive index. (D) Cochallenge infection of mice (*n* = 20) using a 1:1 mixture of the wild type and the Δ*focX* Δ*papX* mutant. Mice were transurethrally inoculated with a 50-μl suspension of a total of 10^8^ CFU/ml. Relative fitness after 48 h postinoculation is presented as a competitive index. Horizontal bars represent the median values for the populations, and the dotted line represents the limit of detection (2 × 10^2^ CFU/g). Statistical differences for cochallenge infections were determined by using a Wilcoxon signed-rank test. ND, not determined.

While P fimbriae have been shown to promote colonization of the kidneys during murine UTI, *papX* has not been confirmed as a fitness factor during *in vivo* infection (46, 47). Instead, PapX has been hypothesized to negatively impact colonization during UTI, as a Δ*papX* mutant showed a slight increase in kidney colonization compared to wild-type CFT073 during an experimental cochallenge in transurethrally inoculated CBA/J mice (33). However, the contribution of PapX during an ascending UTI may be more observable following intraurethral inoculation, since transurethral inoculation places a high number of bacteria directly into the bladder and increases the occurrence of vesicoureteral reflux into the kidneys in the murine model (48). Also, previous work by Lane et al. demonstrated that flagella are a fitness factor for colonization of the bladder and kidneys through independent infections by wild-type CFT073 and a Δ*fliC* mutant following intraurethral inoculation in CBA/J mice (9, 11). Therefore, to assess the contribution of *papX* to *in vivo* colonization, we performed an independent infection of CBA/J mice intraurethrally inoculated with either wild-type CFT073 or the Δ*papX* mutant. Mice were sacrificed at 24 or 48 h, and the bladder, kidneys, and spleen were homogenized and plated to enumerate the bacterial load. We did not observe any significant differences between the wild type and the Δ*papX* mutant in the colonization of the bladder, kidneys, or spleen after either 24 or 48 h (Fig. 7A and B). While we observed high levels of colonization of three spleens infected with the Δ*papX* mutant, we did not have sufficient statistical power to make any conclusions about the role of *papX* in dissemination.

In some cases, a cochallenge model has been shown to be more sensitive than independent challenge for the detection of subtle fitness defects during UTI (49). Thus, to measure relative fitness, CBA/J mice were intraurethrally inoculated with a 1:1 ratio of wild-type CFT073 and the Δ*papX* mutant. We did not observe any statistically significant differences between the wild type and the Δ*papX* mutant in colonization of the bladder, kidneys, or spleen after either 24 or 48 h (Fig. 7C). However, the Δ*papX* mutant tended to have a decrease in colonization of the kidneys after 24 h (*P* = 0.063).

It is possible that FocX can compensate for PapX during UTI and thereby mask fitness defects of the Δ*papX* mutant during infection. Therefore, we performed a cochallenge infection in CBA/J mice transurethrally inoculated with a 1:1 ratio of wild-type CFT073 and the Δ*focX* Δ*papX* double mutant. Mice were sacrificed at 48 h, and the bladder, kidneys, and spleen were homogenized and plated to enumerate the bacterial load. We did not observe any fitness defect in the colonization of the bladder or spleen, but we observed a subtle, but not statistically significant, decrease in colonization of the kidneys (*P* = 0.061) (Fig. 7D).

## DISCUSSION

UPEC strains rely on flagella and fimbriae to successfully ascend and colonize the diverse niches within the urinary tract (7, 10, 50). However, it is not practical for a bacterium to be both motile and adherent simultaneously. Thus, UPEC isolates likely possess mechanisms that mediate a rapid transition between adherent and motile states in response to environmental signals during a UTI. One such mechanism is through the MarR-like protein PapX, which is encoded as part of the *pap* operon and functions as a transcriptional repressor of the flagellar master regulator genes *flhDC* (17, 32, 33). PapX is one member of a subset of 17-kDa MarR-like proteins encoded within fimbrial operons that have been identified in E. coli, and the presence of UPEC strains harboring multiple MarR-like “X” proteins raises the notion that these proteins may function to cooperatively regulate motility (32). However, another “X” gene, *vatX*, has also been identified in strain CFT073 (51). While VatX shares 44% amino acid identity with PapX, neither the deletion nor the overexpression of *vatX* impacted the motility of CFT073 (52). Hence, the study of PapX and its homologs is central to our understanding of UPEC ascension and colonization of the urinary tract.

In this study, we investigated the regulation of *focX* and *papX* gene expression and characterized the effect of encoding multiple PapX homologs on motility. We found that ectopic expression of *focX* or *papX* resulted in comparable inhibition of motility that corresponded with decreased flagellar gene expression (*flhD*, *fliA*, and *fliC*) and reduced flagellin (FliC) production. Therefore, FocX and PapX share the same function as repressors of motility. However, in E. coli, a single bacterium typically expresses one dominant fimbrial type at one time, and since *focX* and *papX* are located within different fimbrial operons, they are likely expressed in an individual bacterium at different times (53, 54). Therefore, during infection, UPEC strains encoding multiple X proteins may be better suited to rapidly repress motility in response to a range of niche-specific cues. Since both P and F1C fimbriae mediate adherence to renal cells, the function of PapX and FocX may be particularly important during kidney colonization (25, 27). We predicted that the production of PapX would improve the success of kidney colonization since the loss of motility improves bacterial attachment. Also, reducing flagellum production likely allows bacteria to better evade host defenses, as the monomeric flagellin FliC is recognized by TLR5 found on host epithelial cells. The activation of TLR5 can lead to the rapid recruitment of neutrophils, inflammation, and, subsequently, bacterial clearance (14, 55). However, we observed during cochallenge in the murine model of ascending UTI that an isogenic Δ*papX* mutant had only a slight decrease in colonization of the kidneys (*P = *0.063). While we predict that FocX shares the same function as PapX, deletion of both X genes did not substantially affect kidney colonization (*P = *0.061). Evaluation of the relative fitness of the Δ*focX* Δ*papX* double mutant instead by intraurethral inoculation may demonstrate a greater effect on colonization. Since there are few studies in the literature exploring the effects of hypermotility on kidney colonization during UTI, further investigation of the function of X genes during infection would broaden our understanding of the role of motility in the development of pyelonephritis.

In this study, we also identified a shared transcriptional start site located 144 bp upstream of the ATG start codon of both *focX* and *papX*, suggesting that *focX* and *papX* can also be regulated independently of their fimbrial operon. A similar transcriptional schema has also been observed for the *papX* homolog *sfaX*, which is transcribed from an independent promoter in addition to being expressed by the *sfa* operon, encoding S fimbriae (43). Thus, dual transcription from two promoters appears to be a conserved regulatory feature of MarR-like proteins encoded within fimbrial operons. In the case of *papX* and *focX*, we found that, in general, the expression of *focX* and *papX* mimicked the expression of their preceding fimbrial operons during *in vitro* culture on LB agar plates, as elevated expression of *papA* was paralleled by an increase in *papX* expression. Overall, the regulation of X genes from an independent promoter may allow for control over flagellar gene expression while independently preserving the appropriate regulation of fimbria production, allowing for fine-tuned transitions in the regulation of adherence and motility factors during ascension from the bladder to the kidneys.

Additionally, an independent proximal promoter may also serve as a site for cross talk between X genes or other transcription factors. Genes encoding MarR-like proteins are commonly autoregulated, where increased protein production creates a negative-feedback loop that inhibits transcription (56, 57). Since FocX and PapX share the same function, we predict that PapX is capable of autoregulation; however, future studies are needed to confirm this regulatory mechanism. Also, we observed that FocX functions as a repressor of *papX*, and this regulation likely occurs at the proximal independent promoter of *papX*, as we did not observe any changes in the expression of the rest of the *pap* operon. However, the overexpression of *focX* had no observable effect on *papX* expression. It is possible that the FocX protein levels present in CFT073/pLX3607 are sufficient for complete repression of *papX*. Thus, increasing the protein concentration of FocX (CFT073/pLX-*focX*) may not translate into a more robust decrease in *papX* transcription. Also, FocX binding may compete with a transcriptional activator of *papX*. For example, the MarR-like protein SlyA promotes gene expression by antagonizing the binding of the nucleoid protein H-NS (58, 59). While there is limited information on the regulators of *papX*, *sfaX* is downregulated by the nucleoid protein H-NS (43). The role of H-NS as a regulator of *papX* has not been well characterized. Interestingly, the transcriptional start site of *sfaX* was located 44 bp downstream from the start site identified for *focX* and *papX*, which may allow H-NS to serve different roles in regulating X genes. For example, H-NS, or another transcription factor, may instead act as a transcriptional activator of *papX*. Therefore, the absence of *focX* may allow for the recruitment of a transcriptional activator of *papX*, while the overproduction of FocX does not directly induce the repression of *papX*.

In contrast, we did not observe that PapX repressed the expression of *focX*, and it is not clear if a required regulatory element is absent from the *focX* promoter or if expression would be better observed under a different condition. In general, cross talk between *focX* and *papX* may serve as a mechanism to limit the negative consequences of extended repression of motility due to excessive X protein production. Also, maintenance of X protein levels appears to be critical for the mechanism behind the repression of motility by PapX and FocX, as we observed in our swimming motility assays that the deletion of *focX* had no effect on motility, while the Δ*focX* Δ*papX* double mutant had an intermediate motility phenotype compared to the hypermotile Δ*papX* mutant. We propose that increased levels of PapX in the *focX* mutant may compensate for the loss of *focX* and thus result in a degree of motility similar to that of the wild type. However, in the double mutant, it may be that the loss of both *focX* and *papX* affects the recruitment of a transcriptional activator of *flhD* or allows another regulator of *flhDC* expression to partially compensate for the absence of X proteins, resulting in an intermediate motility phenotype. The mechanism of PapX and FocX regulation of *flhDC* is not clearly defined. The PapX binding site upstream of *flhDC* does not overlap binding sites of other characterized transcription factors, and the impact of PapX binding within the *flhDC* promoter on downstream access to DNA binding sites for other regulators of *flhDC* is not well characterized (32, 60, 61).

In relation to the *flhDC* promoter, the PapX binding site is centered 220 bp upstream of the *flhDC* transcriptional start site (32, 40). There are numerous transcription factors that have been linked either directly or indirectly to the regulation of *flhDC* expression. For example, the nucleoid-associated protein H-NS promotes *flhDC* expression in part through binding to sites within the *flhDC* promoter as well as repressing the expression of *hdfR*, encoding a transcriptional repressor of *flhDC* (62, 63). Additionally, multiple insertion sequence (IS) elements have been found to integrate into the *flhDC* promoter and affect transcription (42). Therefore, a possible molecular mechanism is that the binding of PapX or FocX affects the accessibility of DNA binding sites within the *flhDC* promoter for other regulatory elements, including other transcription factors and IS elements. Indeed, the IS*5* element in the E. coli K-12 MG1655 strain disrupts the PapX binding site and likely explains why ectopic expression of *papX* in this strain does not impact motility. Thus, preservation of the PapX binding site in the *flhDC* promoter may be more strongly conserved in E. coli strains carrying P, S, or F1C fimbriae than in commensal E. coli isolates. The complex interplay of regulators of *flhDC* expression emphasizes the lengths that bacteria employ to control motility, and in the context of UTIs, MarR-like X proteins may provide a mechanism to mediate fine-tuned coordinated transitions between motility and adherence allowing for better evasion of the host immune system and improved colonization of the upper urinary tract.

## MATERIALS AND METHODS

### Bacterial strains and culture conditions.

Strains used in this study are listed in Table S1 in the supplemental material. Unless otherwise noted, bacteria were cultured in LB medium (10 g tryptone, 5 g yeast extract, 0.5 g NaCl/liter) at 37°C with aeration. UPEC strain CFT073 was isolated from the blood and urine of a patient hospitalized with acute pyelonephritis, and strains F11 and HM69 were isolated from the urine of women experiencing cystitis (64–66). Human urine was collected from at least three women, pooled, filter sterilized, and stored at −20°C in accordance with the recommendations of the University of Michigan Institutional Review Board (approval HUM00004949). When necessary, the following antibiotics were added: ampicillin (100 μg/ml), kanamycin (25 μg/ml), and chloramphenicol (20 μg/ml).

### Mutant and plasmid construction.

The primers used in our study are listed in Table S2 in the supplemental material. The Δ*papX* mutant was constructed as described previously (39). Lambda red-mediated recombineering was used to construct the Δ*focX* and Δ*focX* Δ*papX* mutants. In brief, the chloramphenicol resistance gene (*cat*) was amplified from pKD4 by PCR using EasyA polymerase (Agilent) and primers KO-F and KO-R and then transformed into competent CFT073 cells carrying pKD46 harboring the lambda red operon. Transformed bacteria were cultured at 30°C for 2.5 h, plated on LB agar containing chloramphenicol, and cultured overnight at 37°C (67). The resulting colonies were screened by PCR using *Taq* polymerase (New England BioLabs) and primers scrnF and scrnR for the deletion of *focX*. The same overall strategy was used to construct the Δ*focX* mutants in E. coli F11 and HM69, but the kanamycin resistance gene (*kan*) was instead amplified from pKD3 for transformation.

To construct the Δ*focX* Δ*papX* double mutant, primers KO-F and KO-R, which have homology to both *papX* and *focX*, were used with EasyA polymerase to PCR amplify the *cat* gene from pKD3. The resulting PCR product was transformed into competent CFT073 cells carrying pKD46. Colonies were screened by PCR using *Taq* polymerase and primers scrnF and scrnR to verify the deletion of either *focX* or *papX*. To remove the second X gene, the kanamycin resistance gene was PCR amplified from pKD4 using primers KO-F2 and KO-R2, which flank the first set of primers, and transformed into the competent single mutant strain carrying pKD46. Primers scrnF2 and scrnR2 were used with *Taq* polymerase to screen for the deletion of the second X gene, and the deletion of both *focX* and *papX* was verified by DNA sequencing.

To induce the expression of *papX*, we previously cloned *papX* into the vector pLX3607 under the control of an IPTG (isopropyl-β-d-thiogalactopyranoside)-inducible promoter, referred to as pLX-*papX* (68). A similar strategy was used to control the expression of *focX*. In brief, *focX* was amplified from CFT073 Δ*papX* by PCR using EasyA polymerase and primers pLXfocX-F and pLXfocX-R. Both the resulting PCR product and pLX3607 were digested by NcoI and HindIII, ligated together using T4 DNA ligase (New England BioLabs) to generate pLX-*focX*, and transformed into E. coli Top10 cells (Invitrogen). Transformants were plated on LB medium with ampicillin, and the resulting colonies were screened by PCR using primers scrn_pLX-F and scrn_pLX-R to verify plasmid construction. pLX-*focX* was obtained by using a miniprep kit (Qiagen) from bacterial cultures grown overnight and transformed into CFT073. We utilized “leaky” expression from the P*lac* promoter in the absence of the IPTG inducer to assess the ectopic expression of *papX* or *focX*.

### Swimming motility assay.

Stationary cultures grown overnight were diluted to an OD_600_ of 1.0 and stab inoculated into 0.025% agar motility plates (10 g tryptone, 5 g NaCl, 1.25 g agar/liter). Plates were incubated for 16 to 18 h at 30°C, and the diameter of bacterial spread was averaged as a quantification of swimming motility. Tukey’s multiple-comparison test following analysis of variance (ANOVA) was used for statistical analysis, with the error bars representing the standard deviations.

### qPCR.

Stationary cultures were diluted 1:100 into 25 ml of either LB medium, tryptone medium (10 g tryptone, 5 g NaCl/liter), or sterilized pooled human urine with ampicillin when needed for plasmid maintenance. Strains were cultured at 37°C with aeration until the mid-logarithmic growth phase was reached, and an aliquot was then collected for RNA extraction and treated with a stop solution (95% ethanol [EtOH], 5% phenol) to preserve RNA stability. RNA was also collected from bacteria that were plated on LB agar and incubated at 37°C for 24 h. Plated bacteria were resuspended in 1× phosphate-buffered saline (PBS) (137 mM NaCl, 2.7 mM KCl, 4.3 mM Na_2_HPO_4_, 1.47 mM KH_2_PO_4_/liter [pH 7.4]) and treated with the stop solution. For RNA extraction, bacterial cells were lysed by using 1 mg/ml of lysozyme, and RNA was purified by using the RNeasy kit (Qiagen) according to the manufacturer’s guidelines. RNA samples were treated with DNase I (Ambion), and the removal of genomic DNA was confirmed by PCR using *Taq* polymerase and primers gapA-F and gapA-R. RNA was converted to cDNA by using Superscript III (Invitrogen) and purified by using the PCR cleanup kit (Epoch Life Science). qPCR was conducted by using PowerUP SYBR green (Invitrogen), 4 ng of total cDNA as the template, and the primers listed in Table S2 in the supplemental material. To improve specificity in detecting *papX* and *focX*, qPCR was performed by using a higher annealing temperature and the primers labeled qPCR2 in Table S2. Threshold cycle (*C_T_*) values were normalized to the values for *gapA*, a reference gene, and analyzed by using the ΔΔ*C_T_* method (68). Data are shown as the log_2_ fold changes (FC) of data from three biological replicates. Student’s *t* test was used for statistical analysis.

### Immunoblotting for FliC detection.

Production of FliC was determined by immunoblotting using standardized whole-cell lysates using a previously described method (39). Stationary bacterial cultures were diluted 1:100 in 20 ml of tryptone medium and cultured at 37°C with aeration. Samples were taken at 0.5, 1.5, 3, and 6 h; centrifuged at 1,500 rpm for 10 min to limit the shearing of flagella; suspended in 2× SDS loading buffer (100 mM Tris-Cl [pH 6.8], 4% SDS, 0.2% bromophenol blue, 20% glycerol, 200 mM dithiothreitol [DTT]); and boiled for 10 min at 95°C. Cultures were normalized by the OD_600_ to load individual lanes of a 10% SDS-polyacrylamide gel with similar total protein content. Samples were subjected to SDS-PAGE, followed by transfer to a polyvinylidene difluoride membrane (Immobilon-P; Millipore Corp.). The blot was incubated with a 1:10,000 dilution of rabbit polyclonal antiserum to H1 flagella (Statens Serum Institute, Copenhagen, Denmark), followed by secondary incubation with a 1:20,000 dilution of peroxidase-conjugated goat anti-rabbit immunoglobulin G (Sigma). The Clarity Western ECL substrate kit (Bio-Rad) was used to develop the blot. Data were normalized to the value for a nonspecific band, which served as an additional standard for a loading control.

### 5′ rapid amplification of cDNA ends.

Cultures of Δ*papX* and Δ*focX* strains grown overnight were diluted 1:100 in LB medium and cultured at 37°C to the mid-logarithmic growth phase. An aliquot was collected for RNA extraction and treated with a stop solution (95% EtOH, 5% phenol) to preserve RNA stability. To determine the transcriptional start site of *papX* and *focX*, we used a 5′ rapid amplification of cDNA ends (RACE) kit (Invitrogen) according to the manufacturer’s guidelines, and the primers are listed in Table S2 in the supplemental material. cDNA was inserted into pCR2.1-TOPO via the TOPO TA cloning kit (Invitrogen) and transformed into E. coli Top10 cells (Invitrogen). Transformants were plated on LB with ampicillin, and plasmids were isolated by miniprep and sequenced to determine the *papX* and *focX* transcription initiation sites.

### Cochallenge and independent infections in the mouse model of UTI.

Six- to eight-week-old female CBA/J mice (Jackson Laboratories) were infected as previously described (11, 69, 70). Briefly, bacteria were grown to stationary phase in LB medium and, to induce motility were then diluted 1:50 in fresh LB medium and cultured with aeration at 37°C until an OD_600_ of ∼0.3 was reached. Bacteria were harvested by centrifugation (1,500 rpm), resuspended in sterile PBS, and adjusted by the OD to a final total concentration of 10^7^ CFU/ml. For independent intraurethral infections by E. coli CFT073 and the Δ*papX* construct, mice were inoculated over a 6-s period with a low-dose inoculum (10 µl of 10^7^ CFU/ml) of each strain into the proximal end of the urethra. For cochallenge infections by E. coli CFT073 and the Δ*papX* construct, mice were inoculated intraurethrally by using the same technique but were instead inoculated with a 1:1 mixture of the wild type and the Δ*papX* mutant (10 µl of a total of 10^7^ CFU/ml).

A transurethral infection model was used for cochallenge infections by E. coli CFT073 and the Δ*focX* Δ*papX* construct. Bacteria were grown to stationary phase in LB medium and then harvested by centrifugation (4,000 rpm), resuspended in sterile PBS, and adjusted by the OD to a final total concentration of 10^8^ CFU/ml. Mice were transurethrally inoculated (50 µl of 10^8^ CFU/ml) with a 1:1 mixture of the wild type and the Δ*focX* Δ*papX* mutant. Dilutions of the initial inoculums were plated to verify the input CFU per milliliter.

For all infections, after either 24 or 48 h postinoculation, mice were sacrificed, and the bladder, kidneys, and spleen were removed; homogenized in PBS (GLH homogenizer; Omni International); and plated onto LB agar by using an Autoplate 400 spiral plater (Spiral Biotech). Bacterial counts were enumerated by using a QCount automated plate counter (Spiral Biotech) to determine the output CFU per gram of tissue. Statistically significant differences were determined for independent infections by using a Mann-Whitney test. For cochallenge infections, competitive indices (CI) were calculated as the ratio of mutant to wild-type bacteria in the output over the ratio of mutant to wild-type bacteria in the input inoculum. Statistically significant differences were determined by using a Wilcoxon signed-rank test. All animal protocols were approved by the Institutional Animal Care and Use Committee (IACUC) at the University of Michigan (approval PRO00007111).

## Supplementary Material

Supplemental file 1
