# Supplementary material for: Cross Talk between MarR-Like Transcription Factors Coordinates the Regulation of Motility in Uropathogenic Escherichia coli
Source: Infect Immun. 2018 Nov 20;86(12):e00338-18. doi: 10.1128/IAI.00338-18 (PMC6246914; doi:10.1128/IAI.00338-18)
Supplement: Supplemental file 1 [file zii999092608s1.pdf]

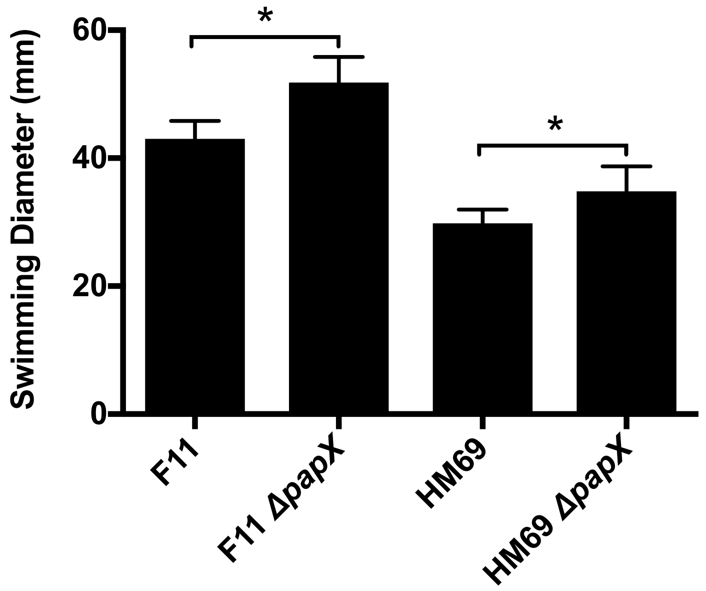

Fig. S1. The loss of *papX* in the cystitis UPEC isolates F11 and HM69 increases motility. Bars represent the average diameter (mm) of swimming motility of bacteria following a 16-18 hr incubation at 30ºC (N=6). The error bars represent the standard deviation, and a Students *t*-test was used for statistical analysis. *, $P < 0.05$

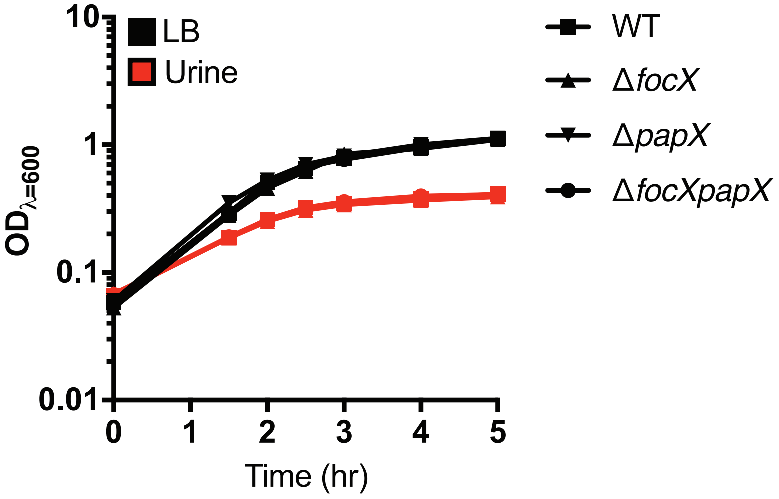

Fig. S2. Growth curves showing similar levels of bacterial growth between CFT073 wild type and the Δ*focX*, Δ*papX*, Δ*focXpapX* constructs. Bacteria were diluted 1:100 from overnight cultures into either fresh pooled, sterilized human urine (red) or LB medium (black) and cultured with aeration at 37°C for 5 hrs. Data represent the OD_{600} measurements taken at different time points.

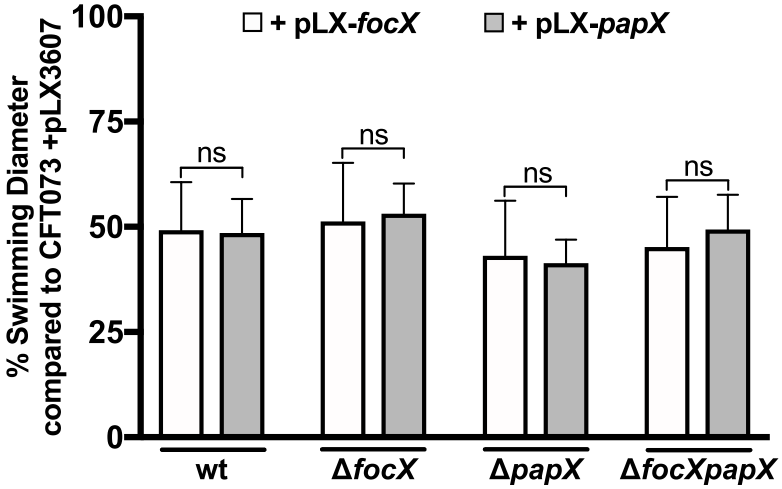

Fig. S3. Expression of either *focX* or *papX* represses motility. Bars represent the average diameter (mm) of swimming motility of CFT073, carrying either pLX-*focX* or pLX-*papX*, compared to CFT073 carrying an empty vector control following 16-18 hr incubation at 30°C. Data represent five biological replicates with the error bars showing the standard deviation. Tukey's multiple comparisons test following ANOVA was used for statistical analysis. *, $P < 0.05$; ns (not significant).

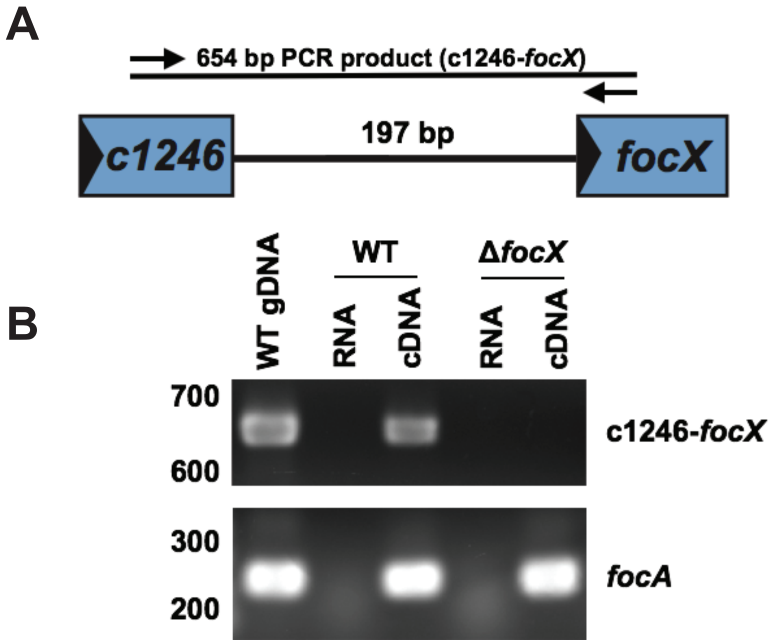

Fig. S4. ***focX* is transcribed as part of the *foc* operon.** (A) Schematic of the c1246 and *focX* genes in the *foc* operon of CFT073. *focX* is located 197-bp downstream of the c1246 allele. (B) PCR using Taq polymerase was performed to determine whether *focX* is transcribed with c1246. Arrows indicate the location of the primers (c1246-Fpcr/focX-Rpcr) used to PCR amplify a 654-bp product (c1246-*focX*) from either CFT073 or Δ*focX* cDNA. Additional primers (papA-Fpcr/papA-Rpcr) were included that amplified a 240-bp product from *focA*, which is included as a positive control confirming cDNA synthesis. CFT073 gDNA acts a positive control, and extracted RNA samples from CFT073 wild type and Δ*focX* were DNase treated and act as a negative control for contaminating gDNA.

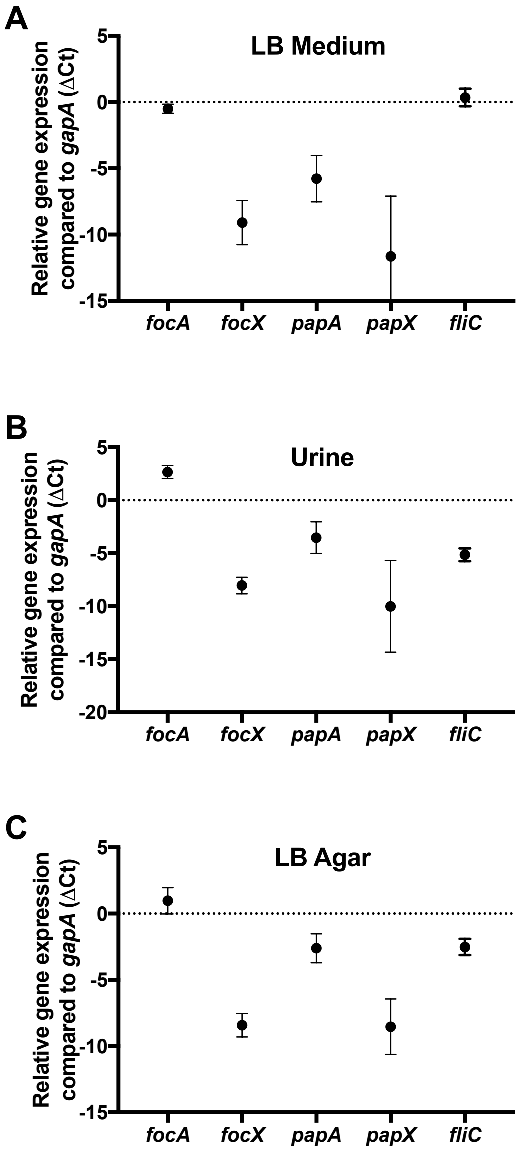

Fig. S5. Relative expression of *foc*, *pap*, and *fliC* compared to *gapA*. qPCR was conducted using cDNA collected from *E. coli* CFT073 wild type cultured to mid-logarithmic growth in either (A) LB medium, (B) sterilized and pooled human urine, or (C) from bacteria cultured on LB agar for 24 hr at 37°C. Data represent the average ΔCt values (reference gene *gapA* - gene of interest (GOI)) of *focA*, *focX*, *papA*, *papX*, and *fliC* with error bars showing the standard deviation. The dashed line at y=0 serves as a reference line for no difference between the Ct values of *gapA* and a GOI.

Table S1. Bacterial strains and plasmids used in this study

Strain	Genotype/Resistance/Use[a]	Source
CFT073	Pyelonephritis isolate (O6:K2:H1)	(1)
ΔfocX	CFT073 *ΔfocX::cat* (Cam^R)	This Study
ΔpapX	CFT073 *ΔpapX::kan* (Kan^R)	(2)
ΔfocXΔpapX	CFT073 *ΔfocX::cat ΔpapX::kan* ($Cam^{R,}$ Kan^R)	This Study
E. coli F11	Cystitis isolate (O6:K2:H31)	(3)
E. coli F11 *ΔpapX*	F11 *ΔpapX::kan* (Kan^R)	This Study
E. coli HM69	Cystitis isolate	(4)
E. coli HM69 *ΔpapX*	HM69 *ΔpapX::kan* (Kan^R)	This Study
**Plasmid**	**Relevant Characteristics**	**References**
pLX3607	IPTG-inducible vector (Amp^R)	(5)
pLX-*focX*	*pLX3607* encoding *focX* at the multiple cloning site (Amp^R)	This Study
pLX-*papX*	pLX3607+*papX,* also known as pDRM001 (Amp^R)	(5)
pKD4	Vector carrying a FRT-flanked *kan* gene (Amp^R, Kan^R)	(6)
pKD3	Vector carrying a FRT-flanked *cat* gene (Amp^R, Cam^R)	(6)
pKD46	Vector carrying phage λ Red recombinase (Amp^R)	(6)

a: cam-chloramphenicol, kan-kanamycin, amp-ampicillin, R-resistant

Table S2. Primers used in this study

Name	Purpose	Sequence (5'-3')
scrnF	Screening by PCR	AGCCCTGTATCTGAGAAAACATGGC
scrnR	Screening by PCR	CACCAGGTCTTTCTGCAACACTACTGC
KO-F	Construction of Δ*focX* and Δ*focX*Δ*papX*	ATCTGTCCGCTGTGCCGGGATATCTCAGTT ATACAGTGTAGGCTGGAGCTGCTTC
KO-R	Construction of Δ*focX* and Δ*focX*Δ*papX*	CTTTATGAGCTGACATCATCAAGATGCGCC AGTAAATGGGAATTAGCCATGGTCC
KO-F2	Construction of Δ*focX*Δ*papX* in CFT073 and Δ*papX* in F11 and HM69	ATGCGCGCTTGTACACAGACAGTGTGTTTC AGTAAGTGTAGGCTGGAGCTGCTTC
KO-R2	Construction of Δ*focX*Δ*papX*	AATGACTTCAAACAGTTCCTGTTCATCATG GGTCAATGGGAATTAGCCATGGTCC9
scrnF2	Screening by PCR	AACGCTGCTCATGATATTGC
scrnR2	Screening by PCR	TCTGCAGGCGCAGCTGGATAAAC
scrnR	Screening by PCR in HM69 and F11 for Δ*papX*	TGAGCTGACATCATCAAGATG
pLXfocX-F	Construction of pLX-*focX*	NNNNCCATGGGGAATAACACAGACACATT
pLXfocX-R	Construction of pLX-*focX*	NNNNAAGCTTTTATGAGCTGACATCATCA AGATG
scrn-pLX-F	Screening by PCR of pLX3607	ATTTGCTTTGTGAGCGGATAAC
scrn-pLX-R	Screening by PCR of pLX3607	CTATCAACAGGAGTCCAAGCTC
gapA-F	qPCR	TGCCTGGCTCCGCTGGCTAA
gapA-R	qPCR	CGCCGCGCCAGTCTTTGTGA
flhD-F	qPCR	TCCGCCTATGTTTCGTCTCGGCATA
flhD-R	qPCR	ACCAGTTGATTGGTTTCTGCCAGC
fliA-F	qPCR	GCGGTATAGAGTGAATTCAC
fliA-R	qPCR	GCGTTTACGCCGCATCCGGC
fliC-F	qPCR	ACAGCCTCTCGCTGATCACTCAAA
fliC-R	qPCR	GCGCTGTTAATACGCAAGCCAGAA
gapA-F	qPCR2	ACCAACTGCCTGGCTCCGCTGGCTAAAGT
gapA-R	qPCR2	CGCGCCAGTCTTTGTGAGACGGGCCATCAA
papA-F	qPCR2	TGGTGCGACAGCAACAGGTGTTTCCTATTT
papA-R	qPCR2	TCGCAACTGCTGAGAAAGCACCTTCTGT
papX-F	qPCR2	TCCATACACTTCCGGCATACAGCG
papX-R	qPCR2	CCCCGCATATCCCTTTCG
focA-F	qPCR2	TGACTCGCAGGTGTCTGCTGGTGCAGGAAT
focX-R	qPCR2	CCGACGTTTGTTGCAGAACCCGCTGCAGAA
focX-F	qPCR2	AGCCCTGTATCTGAGAAAACATGGC
focX-R	qPCR2	CACCAGGTCTTTCTGCAACACTACTGC
fliC-F	qPCR2	TGGTGCGACTGCGGCTACGCTTGATGGTTT
fliC-R	qPCR2	AGTTGCAGCACCAGCATCAGCACCCGTTT
gsp1	5'RACE	ATACCTTCAGATGTCAG
gsp2	5'RACE	ATCCTCCCTGCTATCCATCC

nested_gsp2	5'RACE	CTGAAGGTGACAGACAATGACTCTCC
papA-Fpcr	PCR of *papA*	ACAAATGCGTCTGCTGTCAC
papA-Rpcr	PCR of *papA*	TGGGCCTGAAAAGACAATTC
c1246-Fpcr	PCR of c1246-*focX*	CCGGTGATGACCCCCTGTTA
focX-Rpcr	PCR of c1246-*focX*	TTTGCGCGAATACAGAGTTG

References

1. Mobley, HL, Green, M, Trifillis, AL, Johnson DE, Chippendale GR, Lockatell CV, Jones BD, Warren JW. 1990. Pyelonephritogenic Escherichia coli and killing of cultured human renal proximal tubular epithelial cells: role of hemolysin in some strains. Infect. Immun. 58:1281-1289.

2. Simms AN, Mobley HL. 2008. PapX, a P fimbrial operon-encoded inhibitor of motility in uropathogenic Escherichia coli. Infect Immun 76:4833-41.

3. Stapleton A, Moseley S, Stamm WE. 1991. Urovirulence determinants in Escherichia coli isolates causing first-episode and recurrent cystitis in women. J Infect Dis 163:773-9.

4. Subashchandrabose S, Hazen TH, Rasko DA, Mobley HL. 2013. Draft genome sequences of five recent human uropathogenic Escherichia coli isolates. Pathog Dis 69:66-70.

5. Li X, Rasko DA, Lockatell CV, Johnson DE, Mobley HL. 2001. Repression of bacterial motility by a novel fimbrial gene product. EMBO J 20:4854-62.

6. Datsenko KA, Wanner BL. 2000. One-step inactivation of chromosomal genes in Escherichia coli K-12 using PCR products. Proc. Natl. Acad. Sci. U.S.A. 97:6640-6645.
